# *De novo* assembly and characterization of breast cancer transcriptomes identifies large numbers of novel fusion-gene transcripts of potential functional significance

**DOI:** 10.1186/s12920-017-0289-7

**Published:** 2017-08-29

**Authors:** Vinay K. Mittal, John F. McDonald

**Affiliations:** Integrated Cancer Research Center, School of Biological Sciences, and Parker H. Petit Institute of Bioengineering and Biosciences, Georgia Institute of Technology, 315 Ferst Dr, Atlanta, GA 30332 USA

**Keywords:** Breast cancer, Transcriptome, Fusion-gene, Chimeric transcripts, *de novo* transcript assembly

## Abstract

**Background:**

Gene-fusion or chimeric transcripts have been implicated in the onset and progression of a variety of cancers. Massively parallel RNA sequencing (RNA-Seq) of the cellular transcriptome is a promising approach for the identification of chimeric transcripts of potential functional significance. We report here the development and use of an integrated computational pipeline for the *de novo* assembly and characterization of chimeric transcripts in 55 primary breast cancer and normal tissue samples.

**Methods:**

An integrated computational pipeline was employed to screen the transcriptome of breast cancer and control tissues for high-quality RNA-sequencing reads. Reads were *de novo* assembled into contigs followed by reference genome mapping. Chimeric transcripts were detected, filtered and characterized using our R-SAP algorithm. The relative abundance of reads was used to estimate levels of gene expression.

**Results:**

*De novo* assembly allowed for the accurate detection of 1959 chimeric transcripts to nucleotide level resolution and facilitated detailed molecular characterization and quantitative analysis. A number of the chimeric transcripts are of potential functional significance including 79 novel fusion-protein transcripts and many chimeric transcripts with alterations in their un-translated leader regions. A number of chimeric transcripts in the cancer samples mapped to genomic regions devoid of any known genes. Several ‘pro-neoplastic’ fusions comprised of genes previously implicated in cancer are expressed at low levels in normal tissues but at high levels in cancer tissues.

**Conclusions:**

Collectively, our results underscore the utility of deep sequencing technologies and improved bioinformatics workflows to uncover novel and potentially significant chimeric transcripts in cancer and normal somatic tissues.

**Electronic supplementary material:**

The online version of this article (doi:10.1186/s12920-017-0289-7) contains supplementary material, which is available to authorized users.

## Background

Gene-fusions are a prevalent class of genetic variants that have been implicated in the onset and progression of a variety of cancers [1, 2]. These variants may be generated on the DNA level by genomic rearrangements (e.g., large deletions or insertions, inversions and/or chromosomal translocations [3]). On the RNA level, chimeric transcripts may be generated by co-transcription or transcriptional read-through of neighboring genes [4, 5], or by *trans*-splicing of multiple simultaneously processed pre-mature RNAs from different genes [6, 7]. Recurrent gene-fusions in cancers have often been employed as cancer biomarkers [1, 8] and, in some cases, as potential candidates for targeted gene therapy [9, 10].

In recent years, massively parallel RNA sequencing (RNA-Seq) of the cellular transcriptome has emerged as a promising approach for the identification of previously uncharacterized fusion-gene or chimeric transcripts of potential functional significance [7, 11–15]. In cancer biology, for example, a recent RNA-Seq analysis of 24 primary breast cancer samples uncovered 15 subtype specific fusion-genes that may serve as useful biomarkers of drug sensitivities [16]. In another study, analysis of 89 breast cancer and control samples identified several fusion transcripts involving MAST (microtubule associated serine-threonine) kinase and Notch-family genes that may be drivers of breast cancer onset and/or progression [17].

Currently available computational methods for chimeric transcript discovery such as Tophat-Fusion [18], SnowShoeFTD [19] and FusionSeq [20], typically rely upon reference genome mapping of short (50–75 bp) paired-end reads generated by the sequencing of both ends (5′- and 3′-) of an RNA or cDNA fragment. While these methods are relatively rapid, the results can be ambiguous due to the inherent imprecision associated with genome mapping of short reads [21, 22]. In this study, we take an alternative method of whole transcriptome *de novo* assembly to screen for fusion transcripts in The Cancer Genome Atlas (TCGA) RNA-Seq data of 45 primary breast-cancer and 10 normal-breast tissue samples. We developed an integrated computational workflow to generate significantly longer (>800 bp) contiguous sequences or contigs. These longer contigs not only provide greater accuracy in reference genome mapping but also allow for more reliable identification of splice-variants because longer contigs typically extend across multiple exons [23]. We report here the detection of 1959 chimeric transcripts including 1535 that are specific to the breast cancer samples, 155 that are present only in the normal samples and 269 that are present in both the cancer and normal samples. We found that a number of these fusion transcripts are of potential functional significance including novel fusion-proteins and chimeric transcripts with alterations in their un-translated leader regions (UTRs). A number of breast cancer chimeras mapped to genomic regions devoid of any known genes. Finally, we identified several ‘pro-neoplastic’ chimeric transcripts [24] of potential significance that are suppressed in normal tissue but activated in cancer tissues. Collectively our findings indicate that an unexpectedly large number of chimeric transcripts are present in both cancerous and normal breast tissues and that many of these variants may play a significant role in breast cancer onset and development.

## Methods

### Data acquisition

Forty-five breast adenocarcinoma primary tumors and 10 adjacent normal breast tissue samples were selected from ‘The Cancer Genome Atlas project’ (TCGA) data portal and subsequently RNA-Seq raw data files were downloaded from NCBI-SRA using dbGAP. RNA-Seq data files downloaded in ‘sra’ format were further converted to FastQ format files using the sra-toolkit (https://www.ncbi.nlm.nih.gov/sra/docs/toolkitsoft/). We selected only paired-end reads with high-read coverage to ensure high accuracy in the downstream *de novo* assembly.

### Data analysis

**Fig. 1:**
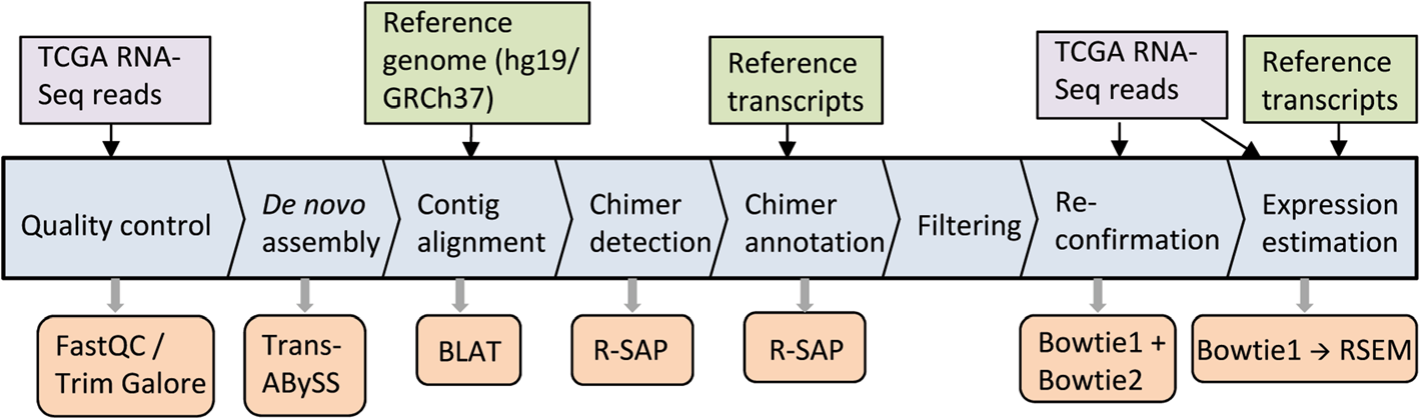
Computational workflow for chimeric transcript discovery. The central blue blocks show the workflow, orange boxes represent the tools and programs integrated with the workflow, purple boxes represent RNA-Seq reads and green boxes represent datasets from the UCSC genome database. RNA-Seq reads (in fastq format) were trimmed and only paired-end reads were used for the assembly process. Assembled contigs (in fasta format) were then aligned to the reference genome and the resulting alignment files (in pslx format) were analyzed by R-SAP to detect potential fusion transcripts. Fusion transcripts were further characterized by comparing alignment coordinates with known reference transcripts (BED format) using R-SAP. Part of the filtering was done by R-SAP internally while additional filtering was done using in-house perl scripts. A re-conformation step includes alignment of RNA-Seq reads to chimeric transcript sequences and also to the reference genome using Bowtie1 and Bowtie 2, respectively. Alignment files (in bam format) resulting from RNA-Seq reads to fusion- transcript sequences were used to estimate the raw read-counts by expectation-maximization using RSEM

For the accurate detection, characterization and quantitative analysis of fusion transcripts using RNA-Seq data, we designed a computational workflow (Fig. 1) that integrates several existing bioinformatics tools including our previously published pipeline R-SAP [25]. The overall workflow is as follows:

#### Data pre-processing

RNA-Seq data may contain low-quality bases due to sequencing errors and fragments of sequencing adapters derived from failed or short cDNA inserts during the library preparation. Such low-quality bases can reduce the efficiency of the assembler and lead to miss-assembly [26]. We, therefore, trimmed low-quality bases (quality score < 20) and sequencing adapters from the 3′-end of the reads using ‘Trim Galore’ [27]. Subsequently the quality of the data was assessed using FastQC [28].

#### Transcriptome assembly

Since a major objective of this study was to detect fusion transcripts where two non-contiguous genomic loci are involved, a reference genome guided assembly approach could not be used. Hence, we performed *de novo* assembly (assembly without the reference genome) using ABySS that is a memory efficient de Bruijn graph construction based short-read assembler [29]. The *de novo* assembly process merges short DNA or RNA sequences that share terminal overlapping bases into a longer contiguous sequence (contig). The length of the terminal overlap or “k-mer length” is a critical parameter for assembly programs. Unlike genomic libraries, where a uniform representation of each base pair can be assumed, non-normalized transcriptome libraries contain a broad range of expressed transcripts and splicing isoforms. Therefore, complete coverage of the transcriptome cannot be achieved at a single k-mer value assembly [30]. To maximize coverage, we adopted previous recommendations [30] and varied the k-mer length from half of the read length up to the full read length in increments of two base pairs at a time. For example, for a library with 50 bp long reads, we performed assembly for k-mer length of 25, 27, … 49. Multiple k-mer assemblies were then merged into a single meta-assembly by using the Trans-ABySS pipeline [30] that combines overlapping contigs by extension and removes duplicate contigs from the assembly.

#### Chimeric transcript detection and filtering

Assembled transcripts were aligned to the human reference genome (hg19, GRCh37) using BLAT (Blast like alignment tool; [31]). BLAT reports independent alignment of different fragments of the RNA sequences and allows long gaps in the alignment that can be representative of introns present in a RNA sequence. We observed the presence of short stretches of homopolymers (poly As and poly Ts) toward the ends of the assembled contigs. Such repeats may affect the overall alignment and may create ambiguous alignments. We therefore trimmed homopolymer repeats as well as other low complexity repeats detected using RepeatMasker (http://www.repeatmasker.org) and Tandem Repeat Finder (http://tandem.bu.edu/trf/trf.html).

For potential chimeric transcript detection, we employed our previously developed pipeline R-SAP [25] that efficiently detects gene-fusion events and filters potential false positives and alignment errors. Alignment files were exported in ‘.pslx’ format from BLAT and were supplied to R-SAP as input for detecting chimeric transcripts. Chimeric transcripts result in fragmented (or split-) alignments where fragments of the chimeric transcripts map to discrete genomic loci. R-SAP detects such alignments and derives the underlying fusion structure using the known gene models. We combined Ensembl and lincRNA (long intergenic non-coding RNA) annotations (available from UCSC genome database) in order to generate a comprehensive set of known gene models. R-SAP characterized each chimeric transcript based upon the genic regions (5’UTR, protein coding sequences [CDS] or 3’UTR) of the reference transcripts intersecting with the genomic loci involved in the chimeric transcript formation.

**Fig. 2:**
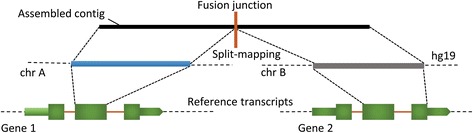
Chimeric transcript detection and characterization by R-SAP. Assembled contigs (black box) representing chimeric transcripts will produce discrete or fragmented alignments (blue and grey boxes) when mapped to the reference genome. It will result in the alignment structure where fragments of the assembled contigs will map to the genomic locations (e.g. chromosome A and chromosome B) underlying the fusion-gene formation. This structure is also called ‘split-mapping’ of the contig. R-SAP detects split-mapping and then compares the alignment coordinate of each fragment with the genomic coordinates of the known reference transcripts (shown in green boxes). Based on the fusion-point mapping (vertical orange bar on the top), R-SAP can determine the transcript regions (such as CDS or UTRs) that are involved in the gene-fusion

Fusion transcripts, representing a fusion-gene event, are very likely to produce discrete alignments to distant or proximate genomic loci. These discrete alignments are also called fragmented- or split-alignments. R-SAP performs the characterization of detected fusion transcripts by associating the fragmented alignments with reference transcripts and categorizes various chimeric transcript structures according to the genic or inter-genic regions to which they map (Fig. 2). We created a comprehensive set of 224,555 reference transcripts by merging Ensembl [32] and lincRNA [33] annotations for hg19 available from the UCSC Genome Browser [34]. These merged annotations were used as the known transcript set for analysis by R-SAP.

Fusion transcripts that were detected and characterized by R-SAP were subjected to additional stringent filtering in order to minimize potential assembly and alignment errors. First, to ensure the validity and significance of the alignment, fusion transcript fragments were required to be at least 25 bp long and to have an alignment identity of >95%. Fusion transcripts with fragments mapping to the same gene were discarded as potential library artifacts. Similarly, fusion-gene events between two paralogous genes (as determined using BioMart for Ensembl genes; [35]) were also discarded because they may potentially represent alignment errors.

Additional potential chimeric transcripts were discarded if either component fulfilled at least one of the following filtering criteria: a) Maps to mitochondrial or Y chromosome; b) Overlaps with genome assembly gaps or maps within 100 k bps of centromere or telomeres (assembly gaps, centromere and telomere coordinates were obtained from UCSC Genome Browser [34]); c) Maps to a genomic region containing ribosomal RNAs (defined by UCSC Genome Browser [34]); d) Has >50% overlap with the genomic low-complexity or simple repeat regions (determined by RepeatMasker track in the UCSC Genome Browser [34]).

**Fig. 3:**
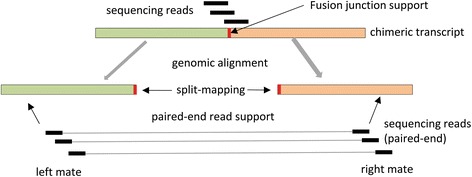
Re-confirmation of chimeric transcripts. In order to remove chimeric transcripts resulting from potential mis-assemblies, we looked for the support for chimeric transcripts (green-orange boxes) in the original RNA-Seq reads (black boxes). RNA-Seq reads were mapped to the chimeric transcripts and reads spanning the fusion-junction (vertical red box) were counted. Reads were also mapped to the reference genome and the occurrence of mate-pairs mapping to the genomic locations underlying the gene-fusions confirmed. We consider a chimeric transcript as ‘confirmed’ if there are at least two reads supporting the fusion-junction and at least two mate pairs supporting the genomic alignment of the chimeric transcript

In order to further filter potentially miss-assembled fusion contigs, we aligned the original RNA-Seq reads to the fusion transcripts using Bowtie [36] in single-end mode and retained only those contigs that had support of at least two sequencing reads at the fusion breakpoint (Fig. 3). We also aligned sequencing reads to the reference genome using Bowtie2 [37] and defined a fusion transcript to be supported by mate-pairs if both mates of the same pair map to the genomic locations involved in the fusion event. We required that each fusion transcript be supported by at least two sets of mate-pairs.

Fusion transcripts are generally considered to be in low abundance in the human transcriptome [38]. Ninety-five percent (52/55) of our samples exceeded the sequencing depth of 100 million reads recommended for optimal detection of low abundance transcripts [30]. In addition, the correlation between the number of reads in the RNA-Seq library and the number of filtered fusion transcripts was insignificant (*R* = 0.24, Student t-test *p*-value >0.05) further indicating our estimates of fusion transcripts are independent of depth of sequencing coverage.

#### Expression quantification

We performed a two-way expression estimation on the filtered set of 1959 chimeric transcripts. First, we estimated the expression (also known as normalized read count) of the reference transcripts (comprised of Ensembl and lincRNA annotation set) that were involved in the chimer transcript formation. Reference transcript sequences were obtained from the UCSC genome database and filtered RNA-Seq reads were mapped using Bowtie. Alignment files were obtained in “bam” format that were sorted using Samtools [39]. Abundance was estimated as expected read counts by using RSEM (RNA-Seq by Expectation Maximization) [40]. Expression values were then normalized using the “Upper quartile normalization” method proposed by Bullard et al. [41]. Expression values of reference transcripts (non-chimers) were used to calculate the fold change of 5′- and 3′- UTR change-associated chimers in cancer samples relative to the normal samples.

In order to determine the relative fusion-read frequency and also the “pro-neoplastic” potential of the nominated chimeric transcripts, we relied upon the expression (or normalized read count) of the chimeric transcript itself rather than the associated reference transcripts. We estimated the expression for each chimeric transcript. RNA-Seq reads were mapped to the assembled contig representing the chimera and read counts were then estimated using RSEM. Read counts were normalized using upper-quartile normalization [41].

Fusion transcript frequency was calculated as percentage of fusion transcript reads relative to total reads that included fusion transcript reads, wild-type 5′-reference gene reads and wild-type 3′-reference gene reads. Expression fold change for pro-neoplastic chimeric transcripts in cancer relative to normal was computed using the average expression values measured across cancer and normal samples.

## Results

### An average of 35 chimeric transcripts per sample were detected in cancerous and normal breast tissue samples analyzed

**Fig. 4:**
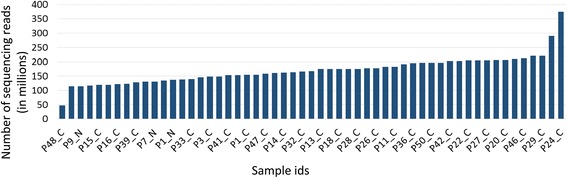
Sequencing coverage distribution across samples. The X-axis displays the 55 breast tissue samples analyzed in the study and y-axis presents the number of reads in millions in each sample

RNA-Seq data for breast cancer and normal breast tissues were downloaded from the TCGA database [42]. The RNA-Seq data (Additional file 1) were generated by sequencing total RNA libraries on the Illumina HiSq2000 system in paired-end mode. The raw data consisted of 50 bp long paired-end reads with an average of 170 million (range 47 million to 374 million; Fig. 4). We selected for analysis only paired-end reads with high read-coverage (45 breast adenocarcinoma primary tumors and 10 normal breast tissue samples) to ensure high accuracy in the downstream *de-novo* assembly. An integrated computational workflow was employed that included the ABySS [29] and Trans-ABySS [30] algorithms to generate long (>800 bp) contiguous sequences or “contigs”. *De novo* assembly (see Methods) of 7.8 billion 50 bp long reads from the 55 RNA-Seq libraries resulted in 12.8 million contigs (an average of 233,615 contigs per sample) with an average length of 860 bps (Additional file 1). The R-SAP algorithm [25] was incorporated into the workflow to identify and characterize chimeric transcripts (Fig. 1). R-SAP follows a hierarchical decision-making procedure to characterize various classes of transcripts such as splice-variants and gene-fusions. Chimeric transcripts (or gene-fusions) are detected by comparing the fragmented reference genome alignments of assembled contigs with well-annotated reference transcripts. R-SAP also applies stringent filtering to limit the potential of false-positive detection (for an independent experimental validation of our pipeline see Additional file 2).

**Fig. 5:**
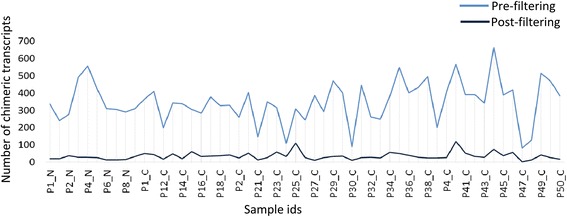
Chimeric transcript distribution across samples before and after filtering. The X-axis displays the 55 breast tissue samples analyzed in this study; the y-axis displays the number of chimeric transcripts per tissue sample. Pre-filtered chimeric transcripts (blue line) are those that were detected by R-SAP while post-filtered chimeric transcripts (black line) are those that were retained after initial filtering, re-confirmation and removal of immunoglobulin (Ig) gene-associated chimers (see Methods for details)

**Fig. 6:**
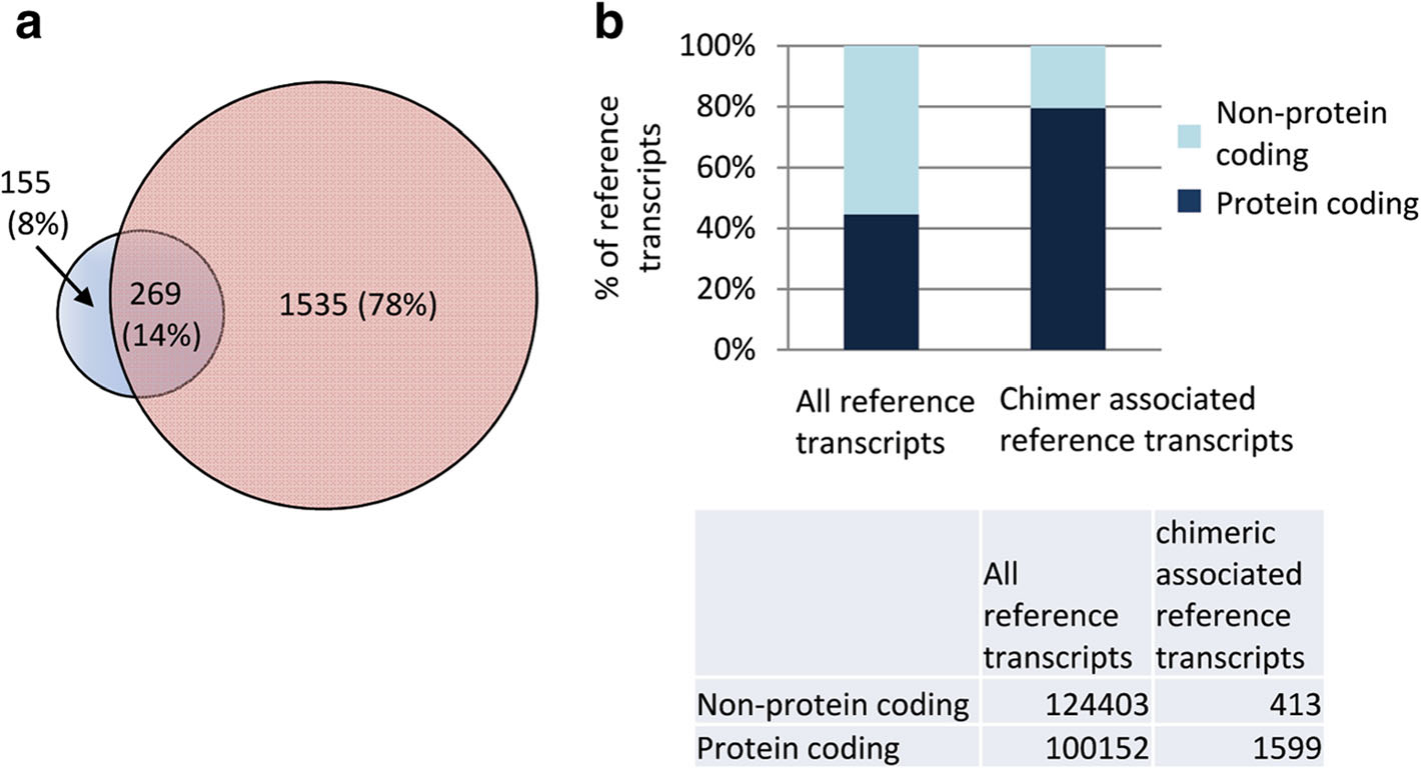
Distribution of fusion and associated reference transcripts. (**a**) Venn diagram representing the distribution of fusion transcripts in 10 normal (blue) and 45 cancer (red) breast tissues. Two-hundred and sixty-nine fusion transcripts were found in both normal and cancer samples. (**b**) Relative distribution of protein coding (black) and non-protein coding (blue) reference transcripts associated with all annotated human transcripts vs. the relative distribution associated with fusion transcripts detected in this study. Table insert displays the total numbers of transcripts in each category

After subjecting the putative chimeric transcripts to a stringent set of filtering criteria (see Methods), 2461 high-confidence chimeric transcripts remained. Of these, nearly 21% were immunoglobulin (Ig) gene fusions likely due to infiltrating T-cells in breast tissue and were excluded from further analysis. After this additional filtering, 1959 chimeric transcripts remained with an average of 35 chimeric transcripts per sample (range 3 to 121) (Fig. 5). We compared chimeric transcripts across all normal and cancer samples by comparing the genomic alignment coordinates of each partner fragment of the chimeric transcript and allowing up to six base pairs to vary around the breakpoint. Out of the 1959 identified chimeric transcripts, 1535 were detected only in the cancer samples, 155 were detected only in the normal samples and 269 were detected in both the normal and cancer samples (Fig. 6a).

### Chimeric transcripts were classified based on structural and functional criteria

A detailed characterization of all chimeric transcripts identified in this study was carried out using the R-SAP algorithm [25] and employing a comprehensive set of 224,555 reference transcripts (Ensembl version 73 and lincRNAs, see Methods). Most (98.82%) of the cancer-specific chimeric transcripts overlapped with at least one reference transcript. Overall 2012 reference transcripts (corresponding to 1917 genes) were associated with chimeric transcripts across all breast cancer samples (Additional file 3). Interestingly, the proportion of protein-coding reference transcripts associated with chimeric transcripts was significantly greater (Fisher’s exact test *p* < 0.0001) than the proportion associated with the entire reference annotation set (Fig. 6b). This suggests that protein-coding transcripts may be preferentially selected in the formation of chimeric transcripts.

**Fig. 7:**
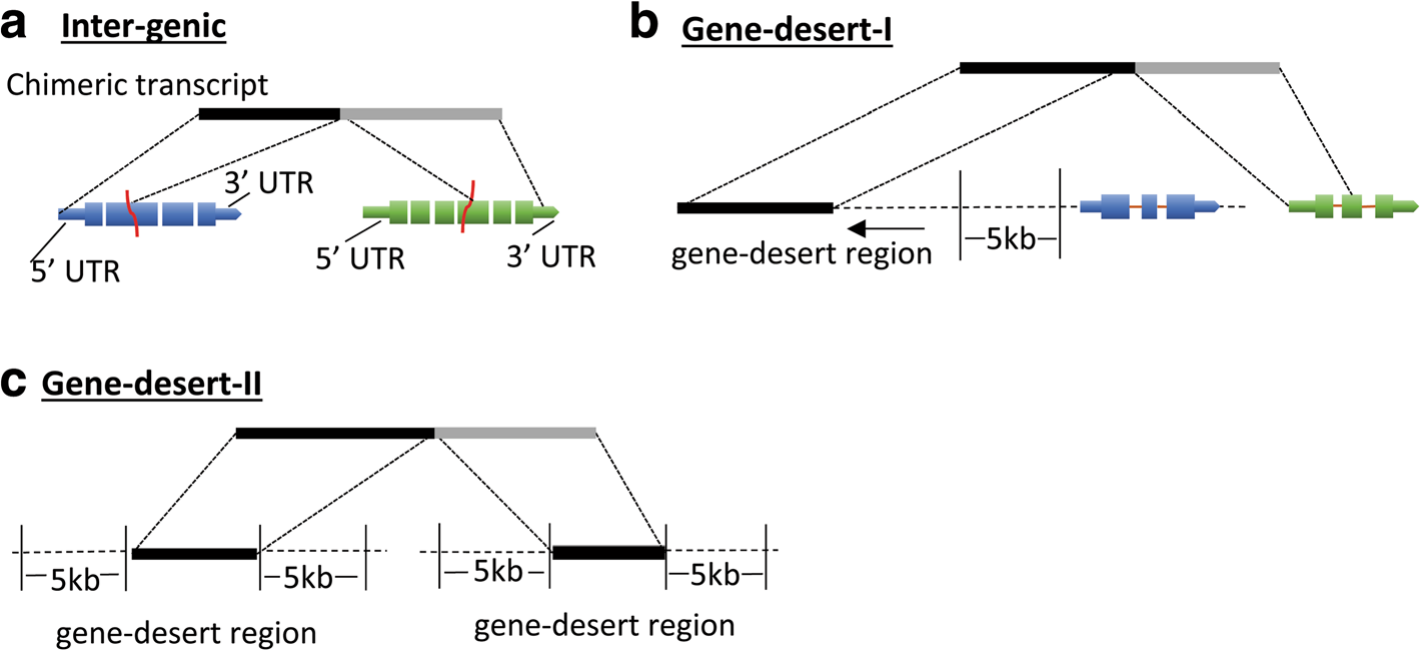
Hierarchical classification system for chimeric transcripts. Chimeric transcripts are depicted as a black-grey box, reference transcripts are represented by blue and green boxes where thick boxes represent open-reading-frames and thin boxes represent 5′ and 3 UTRs. An Inter-genic chimera (**a**) is defined as a chimeric transcript where components map independently to annotated genes; A ‘gene-desert-I’ chimera (**b**) is defined as a chimeric transcript where one component maps to a gene-desert region (black box) while the other maps to an annotated gene (green); A ‘gene-desert-II’ chimera (**c**) is defined as a chimeric transcript where both components map to gene-desert regions. A gene-desert region is defined as the genomic region devoid of any annotated genes within 5 kb of the transcript

**Fig. 8:**
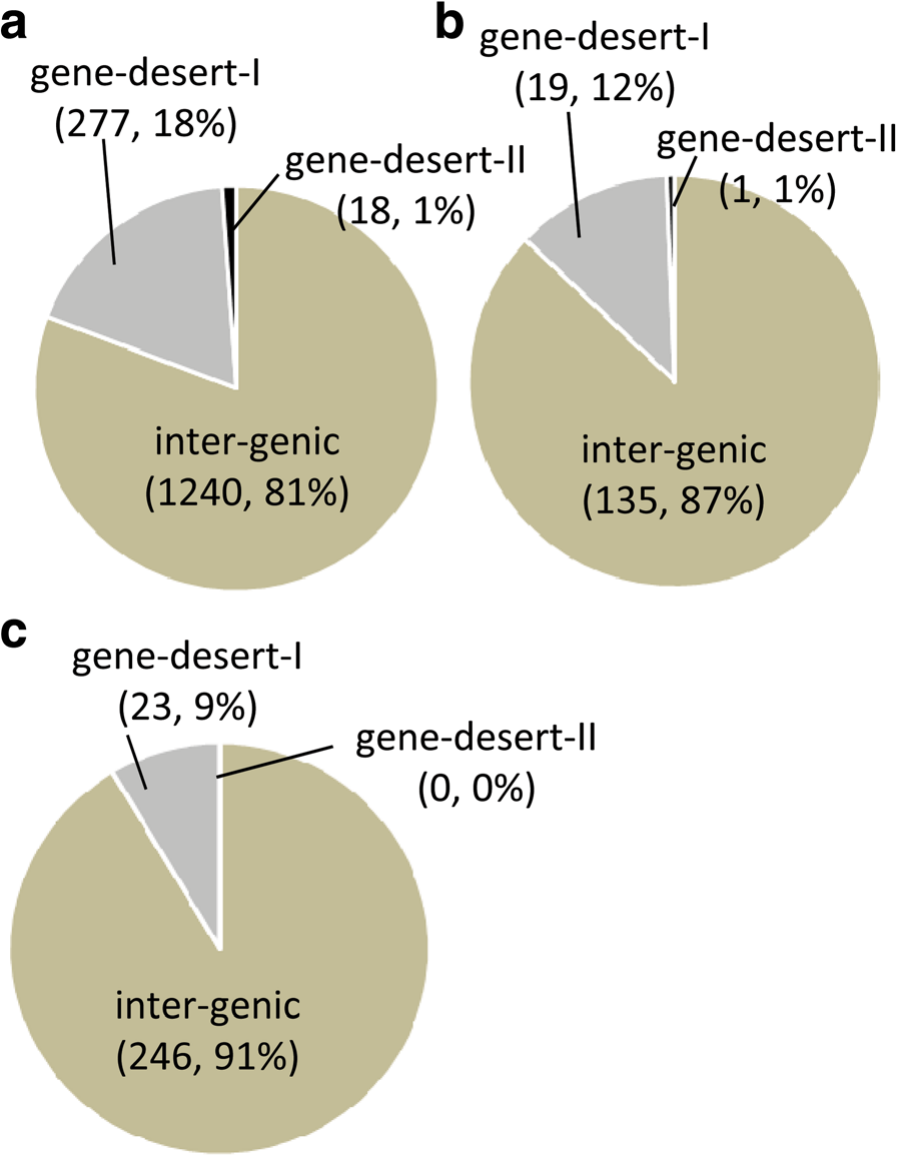
Relative distribution of inter-genic, gene-desert-I and gene desert-II in (**a**) cancer samples, (**b**) in normal tissue samples, and (**c**) in both cancer and normal tissue samples. Classification scheme is described in Fig. 7

To more accurately characterize fusion transcripts and infer potential functional significance, we first established a hierarchical classification system (Fig. 7) where the fusion transcripts were divided into three major classes: inter-genic-where the fusion is composed of two annotated genes; gene-desert I- where the fusion is composed of one annotated gene and a sequence from an un-annotated or “gene-desert” region (lacking any annotated gene within 5 kb); and gene-desert II- where the fusion is comprised of sequences from two distant ‘gene-desert’ regions. Overall, the vast majority (>80%) of fusion transcripts were inter-genic while <18% were gene-desert-I chimers. Only ~1% of the chimers were comprised of two un-annotated transcripts (gene desert-II) (Fig. 8).

**Fig. 9:**
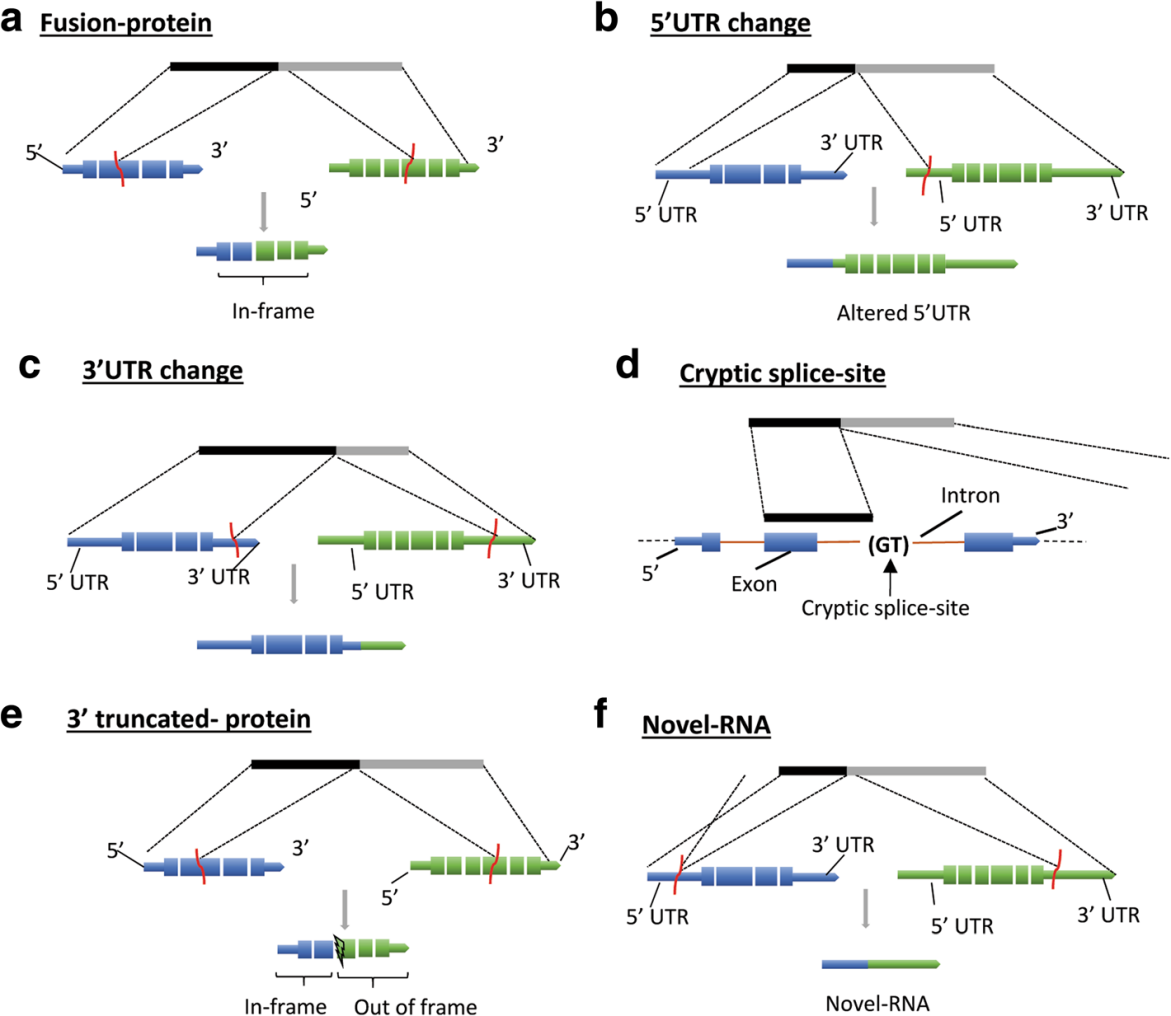
Structure based functional classification of fusion transcripts. Fusion transcripts are represented by black and grey boxes; reference transcripts are represented by blue and green boxes where thick boxes represent exons, gaps represent introns and thin boxes represent the 5′ and 3′ UTRs. Functional classifications are established by comparing the reference genome alignment coordinates of chimeric transcript regions (5’UTR, coding regions or 3’UTR) of the reference transcripts involved in the fusion (spanned by the fusion transcript). **a** Fusion-protein- Fusion of protein coding sequences from two different annotated genes where open-reading frames remain intact; **b** 5′ UTR- Fusion of 5′ UTR from a gene or gene-desert region with protein coding region of another gene keeping the open-reading frame intact; **c** 3′ UTR – Fusion of a 5′ and protein coding region of a gene with the 3′ UTR of another gene or gene-desert region keeping the open-reading frame intact; **d** Cryptic splice-site- A novel splice-variant fusion where the breakpoint lies within a known intron. This group may include inter-genic and gene-desert-I fusions; **e** 3′ truncated-protein- Fusion transcript where the 5′ and coding (in frame) region of one gene is combined with an out-of-frame coding region of another gene or with the 3′ region of a gene-desert region; **f** Novel-RNA- Non-canonical chimeric transcript formation where the potential function of the transcript, if any, is unknown (e.g., 5’UTR-3’UTR fusions). This group also includes out-of-frame truncated fusion-protein transcripts

**Fig. 10:**
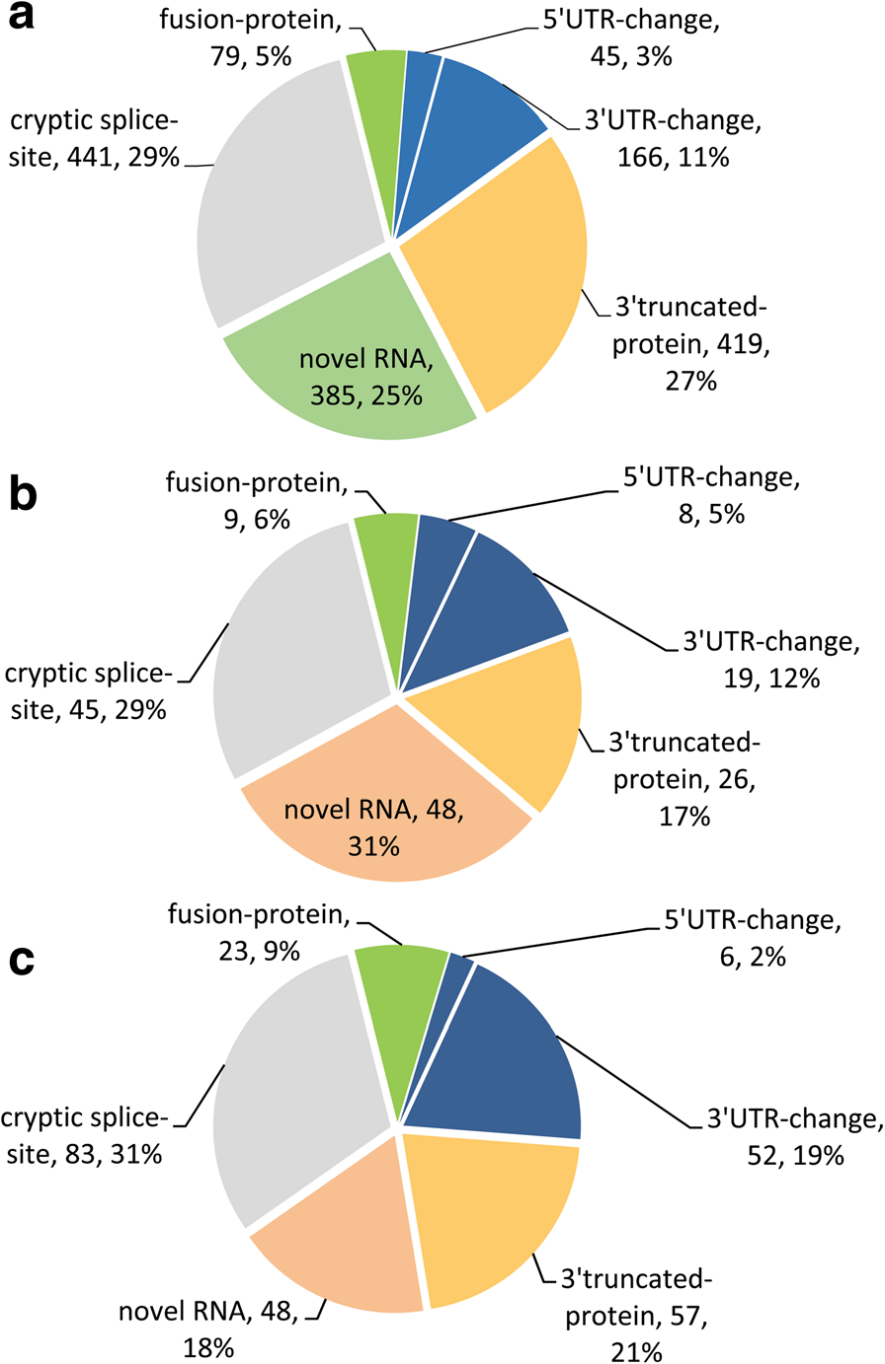
Relative distribution of functional classes of fusion transcripts present. (**a**) Only in cancer tissue samples; (**b**) only in normal tissue samples; and (**c**) in both normal and cancer samples

We further classified the detected fusions into six functional sub-categories (Fig. 9): A) Fusion-protein- fusion transcripts that combine protein coding sequences (CDS) from two different annotated genes while keeping the open-reading frames intact; B & C) 5′ or 3′ UTR- UTR exchange from another gene or gene-desert region in such a way that the original protein-coding region of the fusion remains intact. This group may include inter-genic and gene-desert-I type chimeras (Fig. 7); D) Cryptic splice-site- A novel splice-variant fusion where the breakpoint lies within a known intron. This group may include inter-genic and gene-desert-I chimeras; E) 3′ truncated-protein- The in-frame coding sequence of the upstream (5′) gene in the fusion is partially included (truncated) while the coding region of the 3′ gene is not in frame. This group may include inter-genic and gene-desert I fusions; and F) Novel-RNA- Non-canonical fusion transcript formation where the potential function of the transcript, if any, is unknown (e.g., 5’UTR-3’UTR fusions). This group also includes out-of-frame truncated fusion-protein transcripts. The distribution of the identified chimeras in each of these functional groups is displayed in Fig. 10 and Tables 1, 2 and 3.

**Table 1:** Distribution of cancer specific fusion transcripts across multiple structural and functional classes

	inter-genic	gene-desert-I	gene-desert-II	Total
fusion-protein	79	NA	NA	79
3′ truncated-protein	6	133	NA	419
5′ UTR-change	41	4	NA	45
3′ UTR-change	145	21	NA	166
cryptic splice-site	289	78	NA	367
novel RNA	400	41	18	459
Total	1240	277	18	1535

**Table 2:** Distribution of structural and functional classes for chimers found only in normal tissue samples

	inter-genic	gene-desert-I	gene-desert-II	Total
fusion-protein	9	NA	NA	9
3′ truncated-protein	22	4	NA	26
5′ UTR-change	6	2	NA	8
3′ UTR-change	18	1	NA	19
cryptic splice-site	41	6	NA	47
novel RNA	39	6	1	46
Total	135	19	1	155

**Table 3:** Distribution of structural and functional classes for chimeras found in both normal and in cancer tissue samples

	inter-genic	gene-desert-I	gene-desert-II	Total
fusion-protein	23	NA	NA	23
3′ truncated-protein	53	4	NA	57
5′ UTR-change	6	0	NA	6
3′ UTR-change	52	0	NA	52
cryptic splice-site	33	15	NA	48
novel RNA	79	4	0	83
Total	246	23	0	269

Out of 1535 cancer specific fusions, 5% (79/1535) are fusion-proteins, 3% (45/1535) are 5′ UTR changes and 11% (166/1535) are 3′ UTR changes. The novel-RNAs constitute the most abundant class (30%, 459/1535) of fusion transcripts. The next most frequent class is the 3′ truncated-protein (27%, 419/1535) followed closely by the cryptic splice-site fusions (24%, 367/1535) (Fig. 10a). These relative proportions were generally maintained in the normal specific and overlap class of fusions (Fig. 10b, c).

### Some fusion-protein transcripts recur across the cancer patient samples investigated

**Fig. 11:**
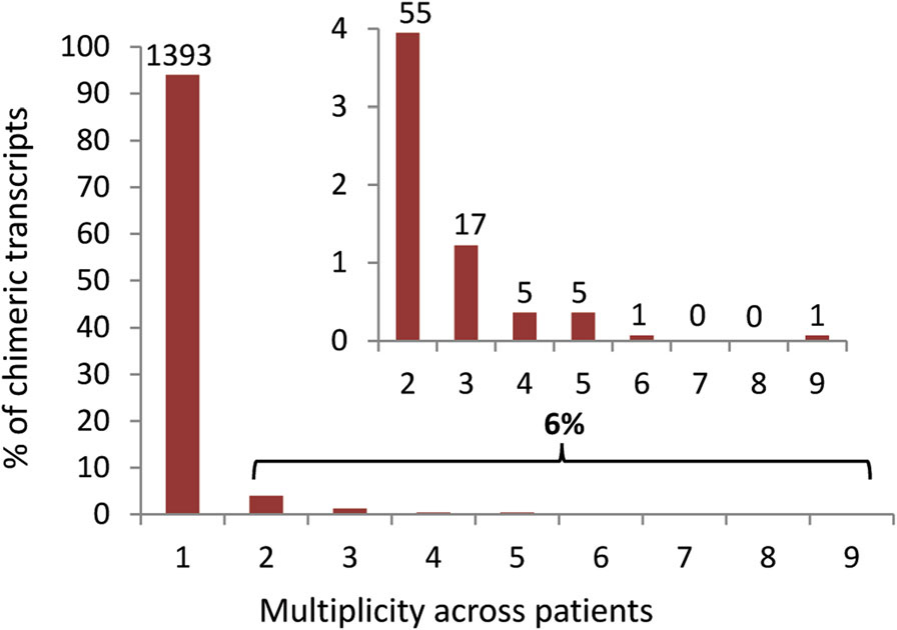
Recurrence of breast cancer associated fusion transcripts across patient samples. Shown is the percentage of all cancer fusion transcripts detected in one or more cancer patient samples. The vast majority of fusion transcripts are specific to individual patients. Inset details the distribution of transcripts found in more than one sample

Although the functional significance of fusion transcripts cannot be unambiguously determined without experimental validation, the recurrence of chimeric transcripts across multiple patients is sometimes taken as tentative indication of biological significance [1]. For example, the *KRI1-ATRX* fusion transcript is the most frequently observed fusion transcript in our dataset (present in nine cancer and one normal samples). It involves a fusion between a partial ORF associated with the *KRI1* (KRI 1 homolog) gene and the DEAD helicase domain (helicase domain containing amino acid sequence D-E-A-D = asp-glu-ala-asp) from the *ATRX* (ATP-dependent helicase ATRX) gene. The DEAD box helicases are a family of proteins involved in ATP hydrolysis dependent DNA and RNA unwinding that, in-turn, regulates RNA expression and its translational efficiency (e.g.*,* [43]. The frequency of recurrent fusion transcripts across cancer samples is shown in Fig. 11 and Table 4.

**Table 4:** Recurrence of chimeric transcripts across cancer samples

Recurrence	Frequency	Percentage
1	1309	93.97
2	55	3.95
3	17	1.22
4	5	0.36
5	5	0.36
6	1	0.07
7	0	0
8	0	0
9	1	0.07
Total	1393	

Recurrence is defined as the number of times a chimeric transcript was found in patient samples. The frequency is defined as the number of chimeric transcripts in each recurrence class

### Seventy-nine cancer-specific fusions encode protein-coding domains where the ORFs are maintained

**Fig. 12:**
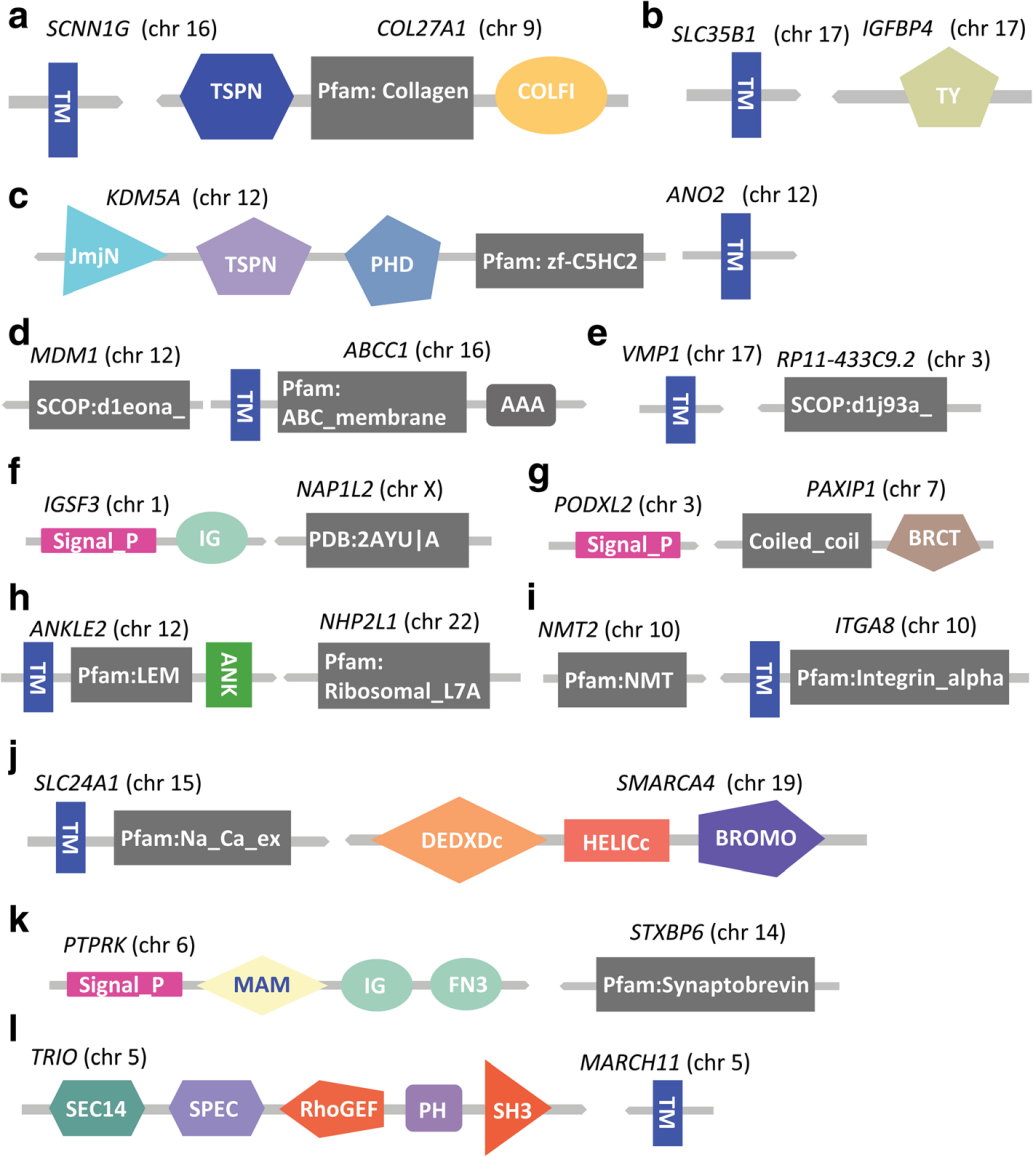
Structure of in-frame gene-fusion mutations resulting in gain of signaling protein domains (trans-membrane and/or signal peptide domains) from another participating gene. Depicted are 12 of 15 detected fusion events where genes were not previously associated with the signaling/TM functions. Gene symbols and corresponding chromosomes (in parenthesis) are shown above each gene fusion structure. Gene symbols are defined as follows: (**a**) *SCNN1G*: Sodium Channel, Non-Voltage-Gated 1, Gamma Subunit; *COl27A1L*: Collagen, Type XXVII, Alpha 1; (**b**) *SLC35B1*: Solute Carrier Family 35, Member B1; *IGFBP4*: Insulin-Like Growth Factor Binding Protein 4; (**c**) *KDM5A*: Lysine (K)-Specific Demethylase 5A; *ANO2*: Anoctamin 2; (**d**) *MDM1*: Mdm1 Nuclear Protein Homolog (Mouse); *ABCC1*: ATP-Binding Cassette, Sub-Family C (CFTR/MRP), Member 1; (**e**) *VMP1*: Vacuole Membrane Protein 1; *RP11-433C9.2*: Clone based putative protein coding gene on chromosome 3; (**f**) *IGSF3*: Immunoglobulin Superfamily, Member 3; *NAP1L2*: Nucleosome Assembly Protein 1-Like 2; (**g**) *PODXL2*: Podocalyxin-Like 2; *PAXIP1*: PAX Interacting (With Transcription-Activation Domain) Protein 1; (**h**) *ANKLE2*: Ankyrin Repeat And LEM Domain Containing 2; *NHP2L1*: NHP2 Non-Histone Chromosome Protein 2-Like 1 (*S. cerevisiae*); (**i**) *NMT2*: N-Myristoyltransferase 2; *ITGA8*: Integrin, Alpha 8; (**j**) *SLC24A1*: Solute Carrier Family 24 (Sodium/Potassium/Calcium Exchanger), Member 1; *SMARCA4*: SWI/SNF Related, Matrix Associated, Actin Dependent Regulator Of Chromatin, Subfamily A, Member 4; (**k**) *PTPRK*: Protein Tyrosine Phosphatase, Receptor Type, K; *STXBP6*: Syntaxin Binding Protein 6 (Amisyn); (**l**) *TRIO*: trio Rho guanine nucleotide exchange factor; *MARCH11*: Membrane-Associated Ring Finger (C3HC4) 11

We identified 79 breast cancer specific fusion transcripts where the fusion occurs within the protein coding regions of the two participating genes and the open-reading frames are maintained (Fig. 10a; Table 1). We analyzed the protein coding domains in these 79 fusion-protein transcripts using SMART (simple modular architecture research tool; [44]). We found that 38% (30/79) of the fusion-protein transcripts contained functional domains for both genes involved in the fusion formation (Additional file 4). Interestingly, 50% (15/30) of these protein coding fusion-transcripts involved the novel joining of a signal peptide (2/15) or a trans-membrane domain (13/15) with a protein coding domain not previously associated with these functional groups. Signal peptide sequences are components of proteins that are normally secreted from cells [45]. Trans-membrane (TM) domains are signaling, transport and subcellular localization components of proteins that are critical to a variety of inter- and intracellular interactions [46–48]. Mutations resulting in the gain or loss of TM domains are known to have a significant effect on cellular functions and molecular interactions [49]. Of the 15 fusions associated with signal peptide/TM domain sequences, 12 are fusions with protein coding sequences (*COL27A1, IGFBP4, KDM5A, MDM1, NAP1L2, NHP2L1, NMT2, PAXIP1, RP11-433C9.2, SMARCA4, STXBP6* and *TRIO*) not previously associated with these signaling functions (genes defined in Fig. 12).

### Fusions that place protein-coding genes under novel regulatory control are frequent in the breast cancer samples investigated

A gene fusion between two different genes often puts one gene (downstream or 3′ partner gene) under the transcriptional regulatory elements (promoter or enhancer) of the other gene (upstream or 5′ partner gene). Such fusion-based regulatory variants have often been associated with the activation of the 3′ proto-oncogene in cancer cells. For example, it has been previously reported that the oncogenic transcription factor *ERG* (ETS-related gene), is up regulated in prostate cancer due to the fusion with the 5′ region of the *TMPRSS2* (trans-membrane protease, serine 2) gene that contains an androgen responsive promoter element [50].

For the 79 fusion-protein transcripts in the cancer samples, we estimated the fold-change in gene expression of the 3′ partner genes involved in the fusion relative to their expression in their normal configurations (i.e.*,* non-chimeric) by comparing the expression of each of the 3′ partners. We used normalized read counts as expression estimates (see Methods) and found that 24% (19/79) of the 3′ partners were associated with a ≥ 2-fold expression increase in cancer for at least one protein coding domain (Additional file 5). Several of the genes involved in these up-regulated fusions have been previously identified as either cancer biomarkers or as potential therapeutic targets. For example, the *B4GALNT2 (*beta-1,4 N-acetylgalactosaminyltransferase 2*)* gene*,* the 3′ partner in the *THRA* (thyroid hormone receptor, alpha)-*B4GALNT2* fusion, has been previously proposed as a prognostic biomarker of breast cancer [51] and is reported to be up regulated in colorectal and metastatic prostate cancer [52, 53]. The *ABCC3* (canalicular multispecific organic anion transporter 2) gene, the 3′ partner in the *MED1* (mediator complex subunit 1)-*ABCC3* fusion, is known to efflux therapeutic compounds resulting in multidrug resistance in cancer cells [54, 55].

**Fig. 13:**
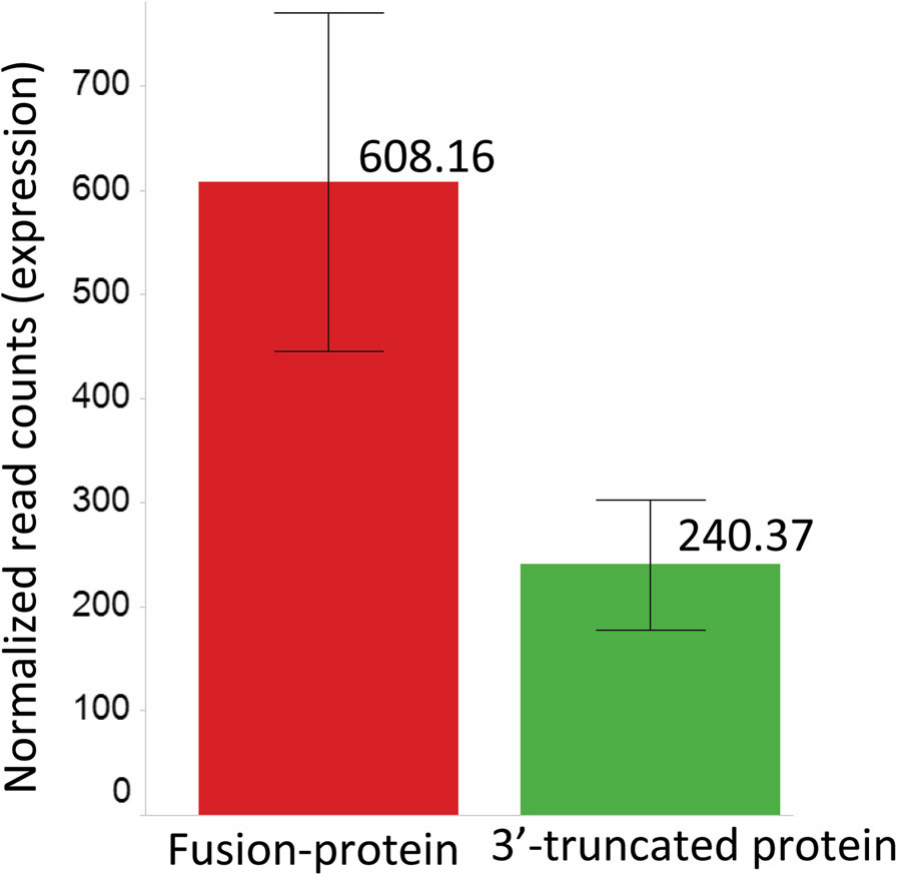
Fusion-protein transcripts have higher expression than 3′-truncated proteins. Chimeric transcripts resulting in 3′-trunctated proteins are likely to produce non-functional transcripts and undergo non-sense mediated decay. Fusion-proteins where the open-reading frame of the participating gene is maintained, on the other hand, are expected to have higher expression than 3′-truncated chimers. The figure shows statistically higher (*p*-value: 0. 005, Student t-test one-tailed) expression for breast cancer specific fusion-protein transcripts (left) than for the 3′-truncated chimeric transcripts (right). Normalized read counts are used as expression estimates. Errors bars shown here are standard errors derived from the expression value distribution across chimer transcript in each category

We also compared the expression of the 79 protein-fusion transcripts with the 419 3′-truncated fusions. The protein-fusions were found to have 2.7-fold higher expression (*p*-value: 0.005; Student t-test one-tailed) than the 3′-truncated fusions (Fig. 13 and Additional file 6) possibly due to non-sense mediated decay (NMD) [56].

Another class of fusions that may be expected to alter patterns of gene expression involves the exchange of 5′ or 3′ un-translated leader regions (UTRs) of intact protein coding sequences. For example, alteration in the poly-A tail attached to 3’UTR and removal of 5′ cap (7-methyle guanosine) may promote mRNA decay and hence overall turnover in the cell [57]. Additionally, fusions involving the exchange of a 5’UTR may place a gene under the control of a novel promoter. For example, chromosomal rearrangements involving UTRs that result in high - level expression of the *ETS* (E26 transformation-specific) gene family members are common events in human prostate cancer [50]. Similarly, changes in the 3’UTR can alter microRNA target binding sites leading to changes in the gene expression. For example, in glioblastoma, the *FGFR3* (fibroblast growth factor receptor 3) gene has been shown to escape regulation by the miR-99a microRNA due to a fusion with the 3’UTR of the *TACC3* (transforming, acidic coiled-coil containing) gene [58].

In our analysis, 14% (211/1535) of the fusions detected in our breast cancer samples consisted of un-disrupted protein coding sequences fused with heterologous UTRs. Nearly 21% (45/211) of these are 5’UTR fusions while 79% (166/211) are fusions with 3’UTRs (Fig. 10a, Table 1). Most (88%, 186/211) of the UTRs were interchanged between two known genes but 12% of the chimers involved the UTRs of known coding sequences with sequences from un-annotated ‘gene-deserts’ regions of the genome (Table 1).

**Fig. 14:**
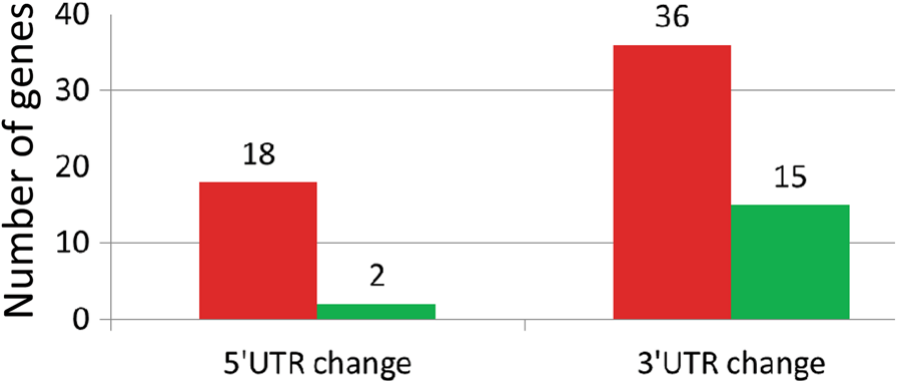
Gene-expression change due to fusion with heterologous UTRs. Chimera formation can result in the altered 5’UTR or 3’UTR while keeping the original ORF intact. Histograms display the number of chimeric transcripts where the protein-coding genes are up-regulated (red) or down-regulated (green) by >2 fold in breast cancer samples relative to the protein-coding genes (native state) in normal breast tissue

We estimated the effects of 5′ and 3′ UTR changes on gene expression by measuring the fold-change in the expression level of each UTR-protein coding gene fusion in the cancer samples relative to the protein-coding gene’s average level of expression in our normal samples (see Methods). The results indicate that 54 of the UTR-protein coding fusion genes are ≥2-fold up regulated relative to their wild-type counterparts in normal cells (Fig. 14; Additional file 7). Several of the up-regulated genes encode transcription factors previously implicated in cancer. For example, the epigenetic transcriptional regulator proteins CBX3 (chromobox homolog 3) and CBX4 (chromobox homolog 4) were up regulated in our cancer samples due to alternative 3’UTRs obtained by gene-fusion. *CBX3* has been previously identified as a potential biomarker for tumor stem cells in osteosarcoma [59], while *CBX4* has been reported to induce hypoxia-mediated activation of *VEGFA* (vascular endothelial growth factor A) and angiogenesis in hepatocellular carcinomas [60]. Another chimeric transcript up regulated in our cancer samples is a fusion of the transcriptional regulator-encoding gene, *RARA* (retinoic acid receptor, alpha), with the 3′ UTR from the *PSME3* (proteasome activator subunit 3) gene. Interestingly, an analogous reciprocal translocation between the *RARA* with *PML* (promyelocytic leukemia) genes has been previously associated with the primary cytogenetic abnormality leading to acute promyelocytic leukemia [61].

In our breast cancer samples, 17 genes were estimated to be ≥2-fold down-regulated due to the fusion with novel UTRs (Table 1; Fig. 14; Additional file 7). For example, a fusion between the *PTEN* (phosphatase and tensin homolog) and the 3′ UTR of the *PIK3C2A* (phosphatidylinositol-4-phosphate 3-kinase, catalytic subunit type 2 alpha) genes resulted in the down regulation of *PTEN >* 2-fold in our cancer samples. *PTEN* is a well-known tumor suppressor gene that displays loss-of-function mutations in many cancers (e.g.*,* [62]).

Other protein coding genes involved in UTR fusions in our cancer samples that have been previously associated with cancer onset and/or progression are the interferon gamma receptor 1 (*IFNGR1*) gene [63], the period circadian clock 2 (*PER2*) gene [64, 65], the chloride intracellular channel 4 (*CLIC4*) gene [66], the sorbin and SH3 domain containing 2 (*SORBS2*) gene [67] and the eukaryotic translation initiation factor 2-alpha kinase encoding (*EIF2AK2*) gene [68, 69].

### A number of detected fusion transcripts include sequences from gene-desert regions of the genome

Previous studies have shown that the human genome is more pervasively transcribed than previously thought [70]. For example, the recent ENCODE (Encyclopedia of DNA Elements; [71]) data release suggests that nearly 80% of the human genome displays transcriptional functionality in a cell type specific manner [72]. Although many of these transcripts are derived from annotated protein-coding genes, others may represent long non-encoding RNAs or other non-encoding regulatory RNAs of currently undetermined function. In our cancer samples, we identified 338 ‘gene-desert’ fusions where either one (319, gene-desert-I) or both components (19, gene-desert-II) of the chimeric transcript maps to the ‘gene-desert’ regions of the genome (Fig. 7; Additional file 8).

**Fig. 15:**
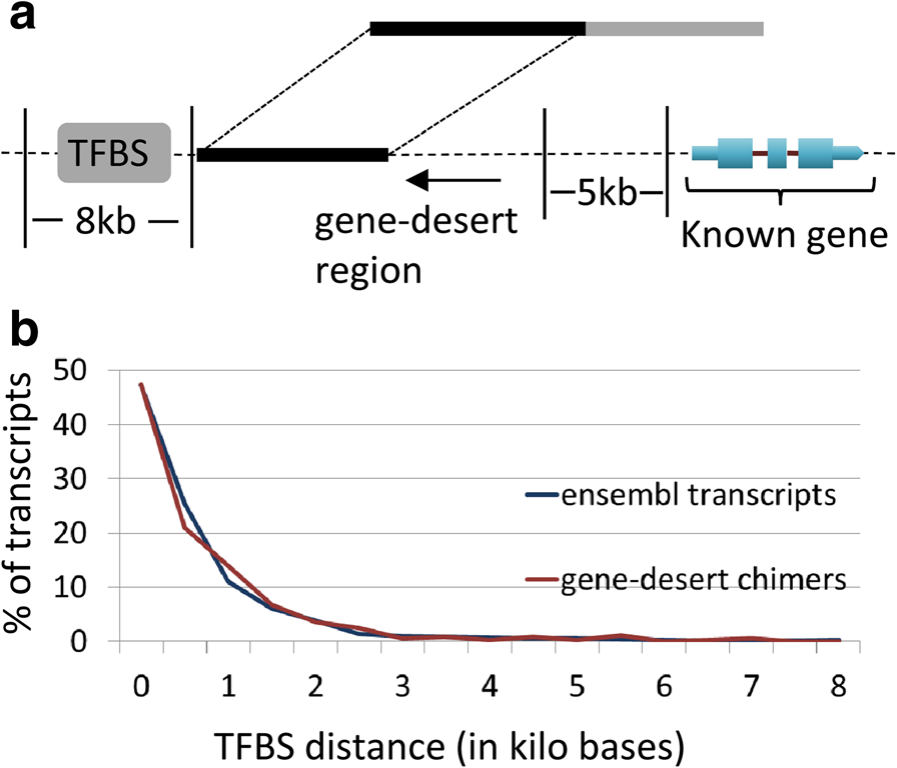
Detection of transcription factor binding sites (TFBS) in proximity to gene-desert regions involved in chimera formation. (**a**) A search was carried out for documented transcription factor binding sites (TFBS; grey box) within 8 kb from gene-desert transcripts (black box) involved in breast cancer gene fusions. (**b**) At least one active TFBS is located within 8 KB of gene-desert transcripts involved in gene-fusions in cancer. The distribution of the locations of TFBS from the gene-desert transcripts (red line) is identical to that associated with annotated reference transcripts (blue line). The x-axis is the distance in kilobases of a TFBS detected from a transcript; the y-axis is the percentage of transcripts with a specific TFBS distance corresponding to X-axis

We obtained transcription factor binding site (TFBS) predictions based on Chip-Seq data from the ENCODE project [71] for five breast or mammary cell lines (HMEC, HMF, MCF-7, MCF10A-Er-Src, T-47D). We then searched for active TFBS in the ENCODE database at positions proximal to gene-desert regions involved in our chimeric transcripts. Since most TFBSs are present within 8 kb of the transcription start site of regulated genes [73], we considered only those TFBSs mapping within 8 kb of the gene-desert transcripts (Fig. 15a). Interestingly, all (100%, 319/319) of the gene-desert regions involved in chimer formation had at least one active TFBS within 8 KB of the transcript. Also, we found that the gene-desert chimeric regions are distributed at distances from TFBS similar to that observed for annotated reference transcripts (Fig. 15b). These findings support the contention that actively transcribed transcripts mapping to gene-desert regions of the genome participate in fusion formation. However, since neither the structure nor the function of transcripts mapping to these gene-desert regions are currently known, the potential functional significance of gene-desert fusions also remains undetermined. Nevertheless, the fact that 9% (28/319) of gene-desert chimeric transcripts involve the fusion of known protein-coding sequences with UTRs from gene-desert regions suggests that at least some of these fusions may represent significant regulatory variants.

### Fusion transcripts are associated with both high and low frequency clones

Because breast cancers, like most solid tumors, are generally polyclonal in make-up [74], RNA-sequencing reads typically represent a mixture of transcripts arising from the various clones comprising the tumor. While higher frequency or predominant clones may make up the bulk of the tumor, a number of lower frequency clones are often also present. To estimate the proportion of the 79 protein fusions associated with high- and low-frequency clones, we computed the frequency of each type of fusion transcript reads relative to the total number of reads detected in the patient samples (see Methods). The results presented in Table 5 (see also Additional file 9) indicate that >50% (43/79) of the identified protein fusions represent ≤10% of total reads in the patient samples indicating that they are likely associated with low-frequency clones. In contrast, 9% (7/79) of the identified protein fusions represent >50% of the identified reads in individual patient tumors indicating that these fusions are associated with high-frequency clones. While the association of protein fusions with high frequency clones is suggestive, the relative frequency of fusions in a tumor sample is not necessarily indicative of relative functional significance. Tumors are a dynamic community of cells where inter-clonal selection is continuously ongoing as new variants arise and/or new environmental challenges (e.g., chemotherapy) are presented to the tumors.

**Table 5:** Distribution of percentage of fusion reads across functional classes in cancer specific chimeric transcripts

chimer functional class	Min%	Max%	Average%	Median%
fusion-protein	0.00360	93.79	16.87	9.16
3′ truncated-protein	0.00034	99.64	15.19	6.54
cryptic splice-site	0.00600	99.83	17.50	4.37
novel RNA	0.00041	99.72	26.23	10.30

Percent of fusion reads was calculated as fusion (chimeric) transcript reads divided by total reads (fusion read count + wild-type (non-fusion) 5′-gene read count + wild-type (non-fusion) 3′-gene read count) (see Methods). RSEM estimated normalized read counts were used. Metric shown in the table were calculated using 1535 breast cancer specific chimer transcripts

### Comparative analysis of fusion transcripts in normal and cancer samples identifies potential pro-neoplastic genes

Comparison of fusion transcripts across all normal and cancer samples was carried out by comparing the genomic alignment coordinates of each partner fragment of the chimeric transcript and allowing up to six base pairs to vary around the breakpoint. Although 88% (1716/1959) of all chimeric transcripts detected were found in the cancer samples and only 12% (243/1959) in the normal samples, this is largely attributable to the disproportionate number of samples examined (45 cancer vs. 10 normal). When the average number of chimers detected per sample is compared, the differences are less dramatic (normal: 24/sample; cancer: 38/sample) albeit still significant (Student’s t-test *p* < 1.05E-03).

The unexpected abundance of chimeric transcripts in normal samples and the fact that the majority of these (> 60%, 269/424; see Fig. 6) were also present in the cancer samples, led us to explore these fusions in more detail. It is possible that at least some of the chimeric transcripts detected in normal tissue may represent “pro-neoplastic” fusions whose cancer-causing potential is at least partially repressed in normal cells (i.e.*,* oncogene expression repressed; tumor suppressor potential amplified). For example, chimeric transcripts of the well-studied chronic myeloid leukemia causing *BCR*-*ABL* (breakpoint cluster region-Abelson protooncogene) fusion gene have been detected at low levels in the blood cells of healthy individuals as well [75]. Similarly, the anti-apoptotic chimeric transcript comprised of the zinc finger genes *JAZF1* (JAZF zinc finger 1) and *JJAZ1* (also known as *SUZ12* or *SUZ12* polycomb repressive complex 2) is highly expressed in nearly 50% of all endometrial stromal sarcomas [76, 77], but has also been detected at low levels in normal endometrial stromal cells as well [24].

**Fig. 16:**
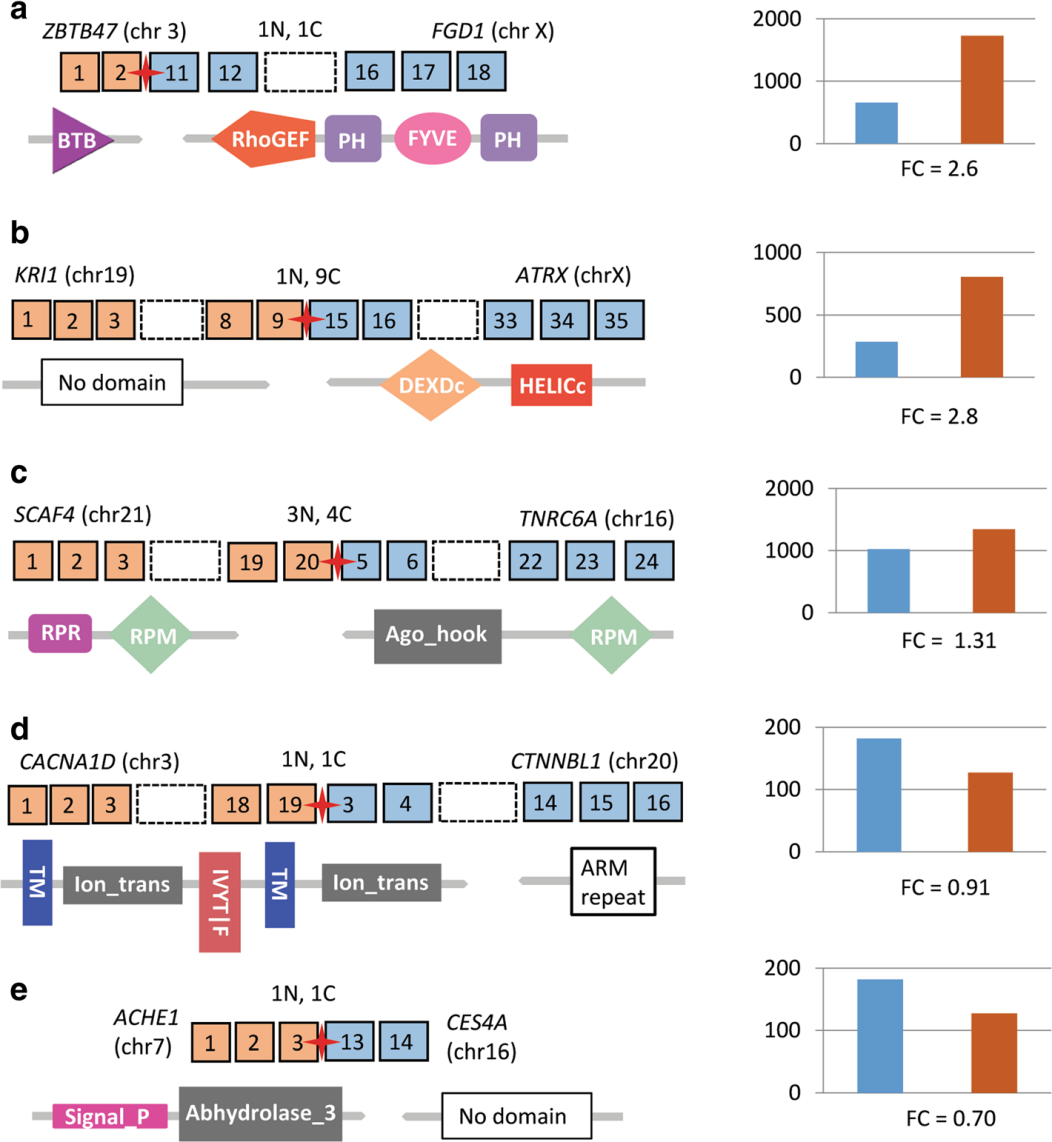
Potential pro-neoplastic gene-fusions that are functionally suppressed in normal breast tissues but activated in cancer tissues. Shown is the structure of five gene-fusions and associated protein domains that we have characterized as potential pro-neoplastic fusions. Square boxes with numbers represent exons (5′ gene: orange, 3′ gene: blue); exons not shown in the figure are represented by a dashed empty box; the red star represents the fusion point for each fusion; gene symbols and (chromosomal location), as well as, the number of each fusion transcript detected in normal (N) and cancer (C) samples is presented above each gene-fusion structure. Protein domains are displayed under each structure. Histograms on the right display average expression levels of the 3′ members of the fusions in their native or parental (pre-fusion) genes in normal samples (blue) and the expression of the fusion transcript in cancer samples (orange) bar. Fold change is shown under each expression plot. All of the 3′ partners of these fusion transcripts have been previously associated with cancer progression (see text for details). (**a**) Z*BTB47-FGD1 =* zinc finger and BTB domain containing 47 gene fused with FYVE, RhoGEF and PH domain-containing protein 1 gene; (**b**) *KRI1-ATRX =* KRI 1 homolog gene fused with ATP-dependent helicase ATRX gene; (**c**) *SCAF4-TNRC6A =* SR-related CTD associated factor 4 gene fused with trinucleotide repeat-containing gene 6A; (**d**) *CACNA1D-CTNNBL1* = calcium channel, voltage-dependent, L type, alpha 1D subunit gene fused with the catenin beta like 1 gene; and (**e**) *ACHE1-CES4A* = acetylcholinesterase 1 gene fused with carboxylesterase 4A gene

We detected 269 chimeric transcripts that were shared between our normal and breast cancer samples. Many of these fusions are associated with moderate- to high-frequency clones (Table 6). For example, four of these shared chimeric transcripts were identified as in-frame fusion-protein coding transcripts of potential pro-neoplastic significance (Z*BTB47-FGD1, KRI1-ATRX, CACNA1D-CTNNBL1*, and *SCAF4-TNRC6A*) (genes defined in Fig. 16; Additional file 10). RNA-Seq reads were mapped to the assembled contigs representing each of these four fusions and read counts were estimated using RSEM (RNA-Seq by Expectation Maximization; [78]) and normalized using upper-quartile normalization [41] (see Methods). Two of the fusions (*ZBTB47-FGD1* and *KRI1-ATRX*) displayed a > 2.5-fold increase in expression in cancer relative to the normal samples (Fig. 16a, b; Additional file 10). Both of these fusions are estimated to be associated with clones in moderately high frequency in their respective tumors based on % of total reads (Additional file 10). A third fusion (*SCAF4-TNRC6A*), also associated with moderately high-frequency clones (Additional file 10), displayed a 1.3-fold increase in expression (Fig. 16c) in the cancer samples while a fourth fusion (*CACNA1D-CTNNBL1*), associated with a lower-frequency clone (Additional file 10), displayed a decrease in expression in the cancer samples (Fig. 16d; Additional file 10).

**Table 6:** Distribution of percentage of fusion reads in nominated pro-neoplastic transcripts in breast cancer

Fusion type	Min%	Max%	Average%	Median%
Pro-neoplastic	0.55318	33.13	12.00	8.93

Percent of fusion reads was calculated as using fusion (chimeric) transcript reads divided by total reads (fusion read count + wild-type (non-fusion) 5′-gene read count + wild-type (non-fusion) 3′-gene read count) (see Methods). RSEM estimated normalized read counts were used. Metrics shown in table are calculated using read counts of pro-neoplastic transcripts in breast cancer samples

In the *ZBTB47-FGD1* fusion transcript, a BTB/POZ domain (BR-C, ttk and bab domain/Pox virus and Zinc finger virus and zinc finger domain) from *ZBTB47* (zinc finger and BTB domain containing 47) is fused with the RhoGEF (a.k.a., the Dbl homologous domain), PH (pleckstrin homology) and FYVE domains from *FGD1*. Interestingly, a previously identified oncogenic fusion gene (*Dbl*) was also found to contain a RhoGEF domain whose over-expression is essential to the *Dbl* gene’s oncogenic potential [79]. Over expression of *FGD1* has also been previously associated with cancer progression in prostate and breast cancer [80]. The 3′ member of the *KRI1-ATRX* fusion (*ATRX*) has been previously associated with childhood neuroblastoma [81] and the 3′ member of the *CACNA1D-CTNNBL1* fusion (*CTNNBL1*), is associated with an anti-apoptotic, tumor suppressive function [82, 83] consistent with its reduced expression in our breast cancer samples.

### Fusion transcripts display breast cancer subtype specificity

Breast cancer is a heterogeneous disease with distinct clinical subtypes [84]. For example, the estrogen receptor negative (ER-), progesterone receptor negative (PR-) and human epidermal growth factor receptor 2 negative (HER2-) (a.k.a., triple negative) sub-type is particularly aggressive and associated with a high risk of metastasis. Previous studies suggest that gene-fusions in breast cancers are often sub-type specific (e.g., [85, 86]). To investigate this question in our dataset, we divided our breast cancer samples into two categories: a) ER+/HER2+ (*n* = 33) and b) triple negative (ER-, PR-, HER2-) (*n* = 12). Consistent with previous reports, we found that the fusions identified in our study were also highly sub-type specific (Table 7). For example, only ≈3% (41/1535 = 0.026) of all of the identified fusion transcripts were detected in both subtype groups (Table 7; Additional file 11). The majority of fusions were associated with the ER+/HER+ sub-group (1052/1535 = 0.68). For the 79 in-frame fusion protein transcripts this preference was even more pronounced (59/79 = 0.75) with only one protein fusion, *LNPEP-ANPEP* (leucyl and cystinyl aminopeptidase- alanyl aminopeptidase, membrane) being shared between the ER+/HER+ and triple negative sub-groups.

**Table 7:** Distribution of chimeric transcripts across breast cancer subtypes

Chimer Functional Class	ER+/HER2+ only	Triple negative only	Common	Total^a^
Fusion-protein	59	18	1	79
3′ UTR change	111	33	11	166
5′ UTR change	31	10	2	45
3′ truncated protein	294	107	9	419
cryptic splice-site	292	133	8	441
novel RNA	265	100	10	385
Overall	1052	401	41	1535

Number of breast cancer specific (not detected in normal tissues) chimeric transcripts from different functional classes in breast cancer subtypes. Two major subtype groups were defined. ER+/HER+ is where patient is either ER+ or HER2+ or both; triple negative where patient is ER-, PR- and HER2-. ^a^Total: Common transcripts are counted twice since they are present in both subtype groups

## Discussion

The oncogenic potential of gene fusions and fusion transcripts was first recognized in malignant hematological disorders and childhood sarcomas [87]. In recent years, the importance of fusions in the onset and progression of a vast diversity of solid tumors has become more widely appreciated. The rapidly growing awareness of the extensiveness and potential importance of fusion transcripts in cancer has been facilitated by the high-throughput transcriptome sequencing of a broad spectrum of cancer types. The Cancer Genome Anatomy Project [88] currently lists well over 1800 fusions identified in >63,000 cancer patient samples and it has been estimated that gene-fusions account for >20% of human cancer morbidity [2].

We present here an integrated computational workflow that not only allows accurate detection of fusion transcripts to nucleotide level resolution but also facilitates detailed molecular characterization and quantitative analysis. We employed this workflow to analyze 55 breast transcriptomes that, to our knowledge, is the first such study to explore global patterns and characteristics of chimeric transcripts in any tumor using a *de novo* assembly approach.

Since the *de novo* assembly approach allows for construction of long contigs capable of traversing multiple exons, we were able to map each gene-associated chimeric transcript to specific genomic loci. Accurate mapping followed by hierarchical structural and functional classification enabled us to systematically infer the potential functional role and biological significance of a number of novel chimeric transcripts. While prior RNA-Seq based studies have focused primarily on the canonical gene fusion structures of fusion-protein and UTR associated alterations, our *de novo* assembly based approach allowed us to explore other classes of fusion structures such as cryptic-splice sites and non-canonical RNA structures. While the accuracy of our pipeline was experimentally validated using a prostate cancer cell line dataset (Additional file 2), the tissue samples used in establishing the TCGA datasets employed in this analysis are not available for experimental confirmation. Thus, although the potential functional impact of many of the chimeric transcripts computationally identified here have yet to be experimentally verified, their widespread occurrence in the breast cancer samples investigated in this study strongly suggests that this class of chimeric transcripts warrants further investigation. In total, we identified 111 novel gene-fusions, 13 of which were detected across multiple patient samples.

Most previously identified gene-fusions in cancer have been associated with oncogene activation [89]. Our findings suggest that gene-fusions can also result in significant down regulation of potentially significant genes. For example, while we identified 54 examples of genes being up regulated in cancer due to fusions with heterologous UTRs, an additional 17 such fusions resulted in a significant down regulation in gene expression including the well-known tumor suppressor gene *PTEN*.

Chimeric transcripts are typically associated with cancer cells but, with notable exceptions (e.g., [90]), their presence in normal somatic cells is often overlooked. In our study, we identified a number of fusion transcripts that are present in both normal and cancer tissues but significantly differentially expressed in these two tissue types. Several of these were identified as potential pro-neoplastic fusions where domains previously associated with oncogenic functions were up regulated in cancer while those previously associated with tumor suppressor functions were down regulated in cancer.

Finally, we detected a large number of chimeric transcripts mapping partially or completely to genomic regions devoid of any known genes (“gene deserts”). We observe that the fusion transcripts involving gene-desert regions can result in the fusion of altered 5′ or 3′ UTRs to known protein-coding genes resulting in significant changes in gene expression. We also detected the fusion of transcripts mapping to two distinct gene-desert regions giving rise to novel RNA structures of currently unknown significance.

## Conclusions

Overall, our *de novo* assembly approach has revealed an unexpected prevalence and diversity of chimeric transcripts in breast cancer tissues. While our results are highly suggestive, we recognize that our conclusions can only be taken as tentative until they are substantiated by experimental validation. While we hope that our findings will stimulate such empirical investigations, we believe our present results underscore the utility of deep sequencing technologies and improved bioinformatic workflows to uncover novel and potentially significant fusion transcripts in cancer and normal somatic tissues.

## Additional files

Additional file 1: Summary statistics on raw and processed RNA-Seq data from the 55 breast samples used in this study. Additional columns contain statistics on assembled contigs, initial and final number of chimeric transcripts after filtering. The first sheet in the excel file contains the data columns and a key describing the data is on the second excel sheet. (XLSX 18 kb)

Additional file 2: Summary of independent validation of fusion detection pipeline. File describes the test dataset, conducted *in silico* experiment and test results. Summary statistics and test results are summarized in tables. (DOCX 20 kb)

Additional file 3: Detailed alignment and annotation information on 1959 filtered chimeric transcripts from 55 samples analyzed in the study. Each chimeric transcript is represented by a unique ID in the first column. Structural and functional classification (as described in the text) information is presented in columns S, T and U. Cells in the gene name columns (‘geneName1’ and ‘geneName2’) with value “none” represent gene-desert regions. The first sheet in the excel file contains the data columns and a key describing the data is on the second excel sheet. (XLSX 684 kb)

Additional file 4: Cancer specific in-frame fusions where at least one protein domain from each (5′ and 3′) of the participating genes is covered by the ORFs involved in the chimera formation. Protein domain names (as defined by SMART database) are present in columns K and T. The first sheet in the excel file contains the data columns and a key describing the data is on the second excel sheet. (XLSX 115 kb)

Additional file 5: Cancer specific in-frame fusions where the 3′ partner gene is up regulated by >2X relative to the intact gene in normal tissue samples. Expression is the normalized RNA-Seq read counts as estimated using RSEM and followed by upper quartile normalization. Expression fold change for the 3′- gene is present in column U. The first sheet in the excel file contains the data columns and a key describing the data is on the second excel sheet. (XLSX 113 kb)

Additional file 6: Expression (normalized read count) for breast cancer specific 79 fusion-protein and 419 3′-truncated protein transcripts. Expression is the normalized RNA-Seq read counts as estimated using RSEM and followed by upper quartile normalization. File contains expression data for breast cancer specific fusion-protein and 3′-truncated protein transcripts only. The first sheet in the excel file contains the data columns and a key describing the data is on the second excel sheet. (XLSX 33 kb)

Additional file 7: Cancer specific chimeric transcripts with fused 5′ or 3′ UTRs and having the ORF of the coding gene intact and displaying >2X change in expression relative to the intact gene’s expression in normal tissue. The first sheet in the excel file contains the key defining column entries. The second sheet contains data for chimeras with a fused 5′ UTR; the third sheet contains data for chimeras with a fused 3′ UTR. For 5′-UTR fusions, the expression fold change for the 3′ partner gene is calculated; for 3′ UTR fusions, the expression fold change for the 5′ partner gene is calculated. The upper portion in each data sheet summarizes the down-regulated genes and the lower portion summarizes the up-regulated genes. (XLSX 27 kb)

Additional file 8: Detailed information for gene-desert-I and gene-desert-II chimeric transcripts. The first sheet of the excel file contains the key defining column entries. The data for cancer specific, normal control and shared chimeric transcripts is presented separately in second, third and fourth sheets, respectively. Cells in the gene name columns (‘geneName1’ and ‘geneName2’) with value “none” represent gene-desert regions. (XLSX 72 kb)

Additional file 9: Fusion read frequency for 79 breast cancer specific fusion-protein transcripts relative to total reads. Expression for fusion transcripts, as well as, those associated wild-type 5′- and 3′- reference transcripts is summarized in the file. Expression is the normalized RNA-Seq read counts as estimated using RSEM followed by upper quartile normalization. Fusion read frequency is shown in column H. The first sheet in the excel file contains the data columns and a key describing the data is on the second excel sheet. (XLSX 19 kb)

Additional file 10: Distribution of fusion reads relative to total reads for pro-neoplastic fusion-protein transcripts in breast cancer samples. File contains fusion specific read counts, as well as, the read counts for wild-type 5′- and 3′- partner reference genes for nominated pro-neoplastic transcripts (in-frame fusion gene transcripts present in both normal and cancer samples) in breast cancer. Expression levels are presented as normalized RNA-Seq read counts as estimated using RSEM and upper quartile normalization. Column J contains relative fusion read frequency as percentage value. The first sheet in the excel file contains the data columns and a key describing the data is on the second excel sheet. (XLSX 12 kb)

Additional file 11: Fusion read relative frequency in breast cancer subtypes. Expression for 79 breast cancer specific fusion transcripts as well as those associated wild-type 5′- and 3- reference genes is summarized in the file. Expression is the normalized RNA-Seq read counts as estimated using RSEM and followed by upper quartile normalization. Fusion transcripts were divided into two breast cancer sub-type groups: ER and/or HER2 positive and triple negative. The first sheet in the excel file contains the data columns and a key describing the data is on the second excel sheet. (XLSX 39 kb)

## Abbreviations

ABCC1: ATP-binding cassette, sub-family C (CFTR/MRP), member 1 gene
ABCC3: ATP-binding cassette sub-family C, member 3 gene
ABL: Abelson protooncogene
ACHE1: Acetylcholinesterase 1 gene
ANKLE2: Ankyrin repeat and LEM domain containing 2 gene
ANO2: Anoctamin 2 gene
ANPEP: Alanyl aminopeptidase, membrane gene
ATP: Adenosine triphosphate
ATRX: ATP-dependent helicase ATRX gene
B4GALNT2: Beta-1,4 N-acetylgalactosaminyltransferase 2 gene
BCR: Breakpoint cluster region
BLAT: Blast-like alignment tool
BTB: BR-C, ttk and bab domain
CACNA1D: Calcium channel, voltage-dependent, L type, alpha 1D subunit gene
CBX3: Chromobox homolog 3
CBX4: Chromobox homolog 4
cDNA: Complementary DNA
CDS: Protein coding sequences
CES4A: Carboxylesterase 4A gene
CLIC4: Chloride intracellular channel 4 gene
COL27A1L: Collagen, type XXVII, Alpha 1 gene
Contig: Contiguous sequence
CTNNBL1: Calcium channel, voltage-dependent, L type, alpha 1D subunit gene
dbGAP: Database of Genotypes and Phenotypes
DEAD: Helicase domain containing amino acid sequence D-E-A-D (asp-glu-ala-asp)
EIF2AK2: Eukaryotic translation initiation factor 2-alpha kinase encoding gene
ENCODE: Encyclopedia of DNA Elements
ER−/+: Estrogen receptor negative or positive gene
ERG: ETS-related gene
ETS: E26 transformation-specific gene
FGD1: FYVE, RhoGEF and PH domain-containing protein 1 gene
FGFR3: Fibroblast growth factor receptor 3 gene
HER2−/+: Human epidermal growth factor receptor 2 negative or positive gene
HMEC: Human microvascular endothelial cell line
HMF: Human mammary fibroblast cell line
IFNGR1: Interferon gamma receptor 1 gene
Ig: Immunoglobulin genes
IGFBP4: Insulin-like growth factor binding protein 4 gene
IGSF3: Immunoglobulin superfamily, member 3 gene
ITGA8: Integrin, alpha 8 gene
JAZF1: JAZF zinc finger 1 gene
JJAZ1: AKA, SUZ12 polycomb repressive complex 2 gene
KDM5A: Lysine (K)-specific demethylase 5A gene
K-mer: All the possible subsequences (of length k) from a read obtained through DNA-seq
KRI1: KRI 1 homolog gene
lincRNA: Long intergenic non-coding RNAs
LNPEP: Leucyl and cystinyl aminopeptidase gene
MARCH11: Membrane-associated ring finger (C3HC4) 11 gene
MAST: Microtubule associated serine-threonine gene
MCF10A-Er-Src: Michigan Cancer Foundation-10A, mammary, non-tumorigenic epithelial inducible cell line, containing a derivative of the Src kinase oncoprotein fused to the ligand-binding domain of the estrogen receptor
MCF-7: Michigan Cancer Foundation-7, breast cancer cell line
MDM1: Mdm1 nuclear protein homolog (mouse) gene
MED1: Mediator complex subunit 1 gene
NAP1L2: Nucleosome assembly protein 1-like 2 gene
NCBI-SRA: National Center for Biotechnology Information-Sequence Read Archive
NHP2L1: NHP2 non-histone chromosome protein 2-like 1 (*S. cerevisiae*) gene
NMD: Nonsense mediated decay
NMT2: N-myristoyltransferase 2 gene
ORF: Open reading frame
PAXIP1: PAX interacting (with transcription-activation domain) protein 1 gene
PER2: Period circadian clock 2 gene
PIK3C2A: Phosphatidylinositol-4-phosphate 3-kinase, catalytic subunit type 2, alpha gene
PML: Promyelocytic leukemia gene
PODXL2: Podocalyxin-like 2 gene
POZ: Pox virus and Zinc finger virus and zinc finger domain
PR−/+: Progesterone receptor negative or positive gene
PSME3: Proteasome activator subunit 3 gene
PTEN: Phosphatase and tensin homolog gene
PTPRK: Protein tyrosine phosphatase, receptor type, K gene
RARA: Retinoic acid receptor, alpha gene
RNA-Seq: Sequencing RNA
RP11-433C9.2: clone based putative protein coding gene on chromosome 3 gene
R-SAP: RNA-Seq analysis pipeline
RSEM: RNA-Seq by Expectation Maximization
SCAF4: SR-related CTD associated factor 4 gene
SCNN1G: Sodium channel, non-voltage-gated 1, gamma subunit gene
SLC24A1: Solute carrier family 24 (sodium/potassium/calcium exchanger), member 1 gene
SLC35B1: Solute carrier family 35, Member B1 gene
SMARCA4: SWI/SNF related, matrix associated, actin dependent regulator of chromatin, subfamily A, member 4 gene
SMART: Simple modular architecture research tool
SORBS2: Sorbin and SH3 domain containing 2 gene
STXBP6: Syntaxin binding protein 6 (Amisyn) gene
T-47D: Breast epithelial metastatic cell line
TACC3: Transforming acidic coiled-coil containing gene
TCGA: The cancer genome atlas
TFBS: Transcription factor binding site
THRA: Thyroid hormone receptor, alpha gene
TM: Transmembrane
TMPRSS2: Transmembrane protease, serine 2 gene
TNRC6A: SR-related CTD associated factor 4 gene fused with trinucleotide repeat-containing gene 6A
TRIO: Trio Rho guanine nucleotide exchange factor gene
UCSC: University of California, Santa Cruz
UTR: Untranslated leader regions
VEGFA: Vascular endothelial growth factor A
VMP1: Vacuole membrane protein 1 gene
ZBTB47: Zinc Finger and BTB Domain Containing 47
