# Supplementary material for: De novo assembly and characterization of breast cancer transcriptomes identifies large numbers of novel fusion-gene transcripts of potential functional significance
Source: BMC Med Genomics. 2017 Aug 29;10:53. doi: 10.1186/s12920-017-0289-7 (PMC5575902; doi:10.1186/s12920-017-0289-7)
Supplement: Supplementary file 2 — Summary of independent validation of fusion detection pipeline. File describes the test dataset, conducted in silico experiment and test results. Summary statistics and test results are summarized in tables. (DOCX 20 kb) [file 12920_2017_289_MOESM2_ESM.docx]

**Additional File 2. Summary of independent validation of fusion detection pipeline.**

In order to test the validity of our pipeline's workflow, we downloaded RNA-Seq data using Short Read Archive (accession: SRR2147349) (Olsen, JR, et al. 2016. BMC Cancer 16:377, doi: 10.1186/s12885-016-2453-4) derived from a well-studied prostate cancer cell line (VCaP) for which a number of fusions have been previously experimentally identified. The RNA-seq data available for this cell line displays similar read coverage, paired-end reads, similar library chemistry and protocol and was obtained using a similar sequencing instrument. Our goal was to see if our pipeline could computationally identify fusions from the RNA-seq dataset that have been previously experimentally verified. The results demonstrate that our pipeline successfully identified the two fusion variants (*TMPRSS2-ERG* and *SPOCK1-TBC1D9B*) that have previously been experimentally detected in VCaP cells (*TMPRSS2-ERG*: see Mertz, KD, et al. 2007. Neoplasia 9(3): 200-206, PMC 1838578; *SPOCK1-TBC1D9B:* see Maher, CA, et al. 2009. Proc Natl Acad Sci, USA 106(30):12353, PMC 2708976).

**Summary statistics on raw and processed RNA-Seq data.** Data are shown for the prostate cancer cell-line, VCaP (SRA accession Id: SRR2147349), used for the *in silico* validation of the fusion-detection pipeline.

| Total reads | 54261114 |
| --- | --- |
| Reads after trimming | 54179042 |
| Total assembled contigs | 696392 |
| Average contig length | 413 bp |
| Chimeric transcripts(unfiltered) | 124 |
| Chimeric transcripts(filtered) | 20 |
| Immunoglobin mapped chimers | 3 |
| Final filtered chimers | 17 |
| average chimer contig length | 792 bp |

**Distribution of functional classes for chimers found in VCaP cell-line.**

| fusion-protein | 2 |
| --- | --- |
| 5’UTR-change | 1 |
| 3’UTR-change | 3 |
| 5-truncated-protein | 4 |
| novel RNA | 4 |
| cryptic-splice-site | 4 |
| **Total** | **18** |
